# Paradox of atropine in myopia control: exploring dose-dependent efficacy, rebound effects, and optimal risk-benefit balance–a perspective

**DOI:** 10.3389/fpubh.2026.1878312

**Published:** 2026-06-24

**Authors:** Ya-zhen Wu, Yuan-yuan Chen

**Affiliations:** Department of Ophthalmology, Xi'an People's Hospital (Xi'an Fourth Hospital), Xi'an, Shaanxi, China

**Keywords:** atropine, axial length, dose-dependent efficacy, myopia control, personalized treatment, public health policy, rebound effect, risk-benefit assessment

## Abstract

The global surge in myopia prevalence, particularly the rising incidence of high myopia and its associated blinding complications—including myopic maculopathy, retinal detachment, and glaucoma—has transformed this condition into a pressing public health priority. Atropine, the most extensively studied pharmacological intervention for myopia control, now stands at a critical crossroads. The shift from high-concentration to low-concentration formulations has markedly improved tolerability, yet a fundamental paradox persists: the dose that minimizes side effects and rebound may be insufficient to halt axial elongation in children at greatest risk. This perspective article argues that atropine therapy faces an “impossible triangle” of dose-dependent efficacy, rebound magnitude, and tolerability, which precludes any single-concentration solution applicable to the entire pediatric population. We propose a redefinition of the risk–benefit assessment framework, moving beyond exclusive reliance on refractive error toward a multidimensional evaluation centered on axial length control, age at treatment initiation, and environmental factors such as outdoor activity. An integrated, stepped-titration strategy that combines pharmacological, optical, and behavioral interventions is advocated. Looking ahead, we call for the development of a multicentre, real-world evidence base with mandatory long-term washout data, which is essential to inform personalized, public-health-oriented myopia management aimed not at temporary dioptric stabilization but at the durable prevention of blindness.

## 1 Introduction

### 1.1 Clinical and public health divide in myopia

The global rise in myopia prevalence has redefined the condition as a significant public health challenge (1). A crucial distinction separates simple school-age myopia from pathological myopia. Nevertheless, the long-term structural implications of axial elongation may not always receive sufficient emphasis in routine refractive management and public health discussions (2). Whereas school-age myopia is primarily managed as a refractive condition, pathological myopia constitutes a progressive structural disease associated with irreversible retinal and choroidal degeneration (3, 4). Simple myopia primarily represents a refractive anomaly that can be corrected with spectacles or contact lenses. From a public health perspective, however, the focus should shift beyond refractive error measured in diopters. The principal driver of irreversible visual impairment is excessive axial elongation of the eye (5). Even a single millimeter of additional axial growth proportionally increases the lifetime risk of sight-threatening complications, including myopic maculopathy, retinal detachment, glaucoma, and cataract (6). Consequently, the ultimate therapeutic objective extends beyond refractive neutralization to the suppression of progressive globe elongation. This reframes intervention assessment from a matter of visual convenience to a medical imperative centered on the long-term preservation of retinal and optic nerve integrity.

### 1.2 Paradigm shift from high-concentration to low-concentration atropine

The pharmacological history of atropine for myopia control illustrates a deliberate therapeutic evolution, driven by the need to reconcile efficacy with tolerability (7). Early regimens using high-concentration atropine (0.5%−1.0%) provided robust evidence of slowed axial elongation, thereby establishing a proof of concept (8). However, these doses induced marked cycloplegia with associated near-vision impairment, manifesting as disabling photophobia and near-vision blur (7, 8). Such functional impairments hindered visual learning and daily activities, rendering long-term use unacceptable for most children (7, 8). These drawbacks motivated a paradigm shift toward low-concentration formulations. The guiding principle was to titrate the dosage downward and identify a minimum effective concentration that could separate the therapeutic action on scleral remodeling from the incapacitating antimuscarinic side effects. The Atropine for the Treatment of Myopia (ATOM) trials systematically explored this therapeutic window (7, 8). The ATOM 2 study brought 0.01% atropine to global prominence, reporting meaningful slowing of myopia progression together with minimal pupil dilation, negligible accommodative loss, and substantially improved tolerability (7).

### 1.3 Articulating the core paradox

**Figure 1:**
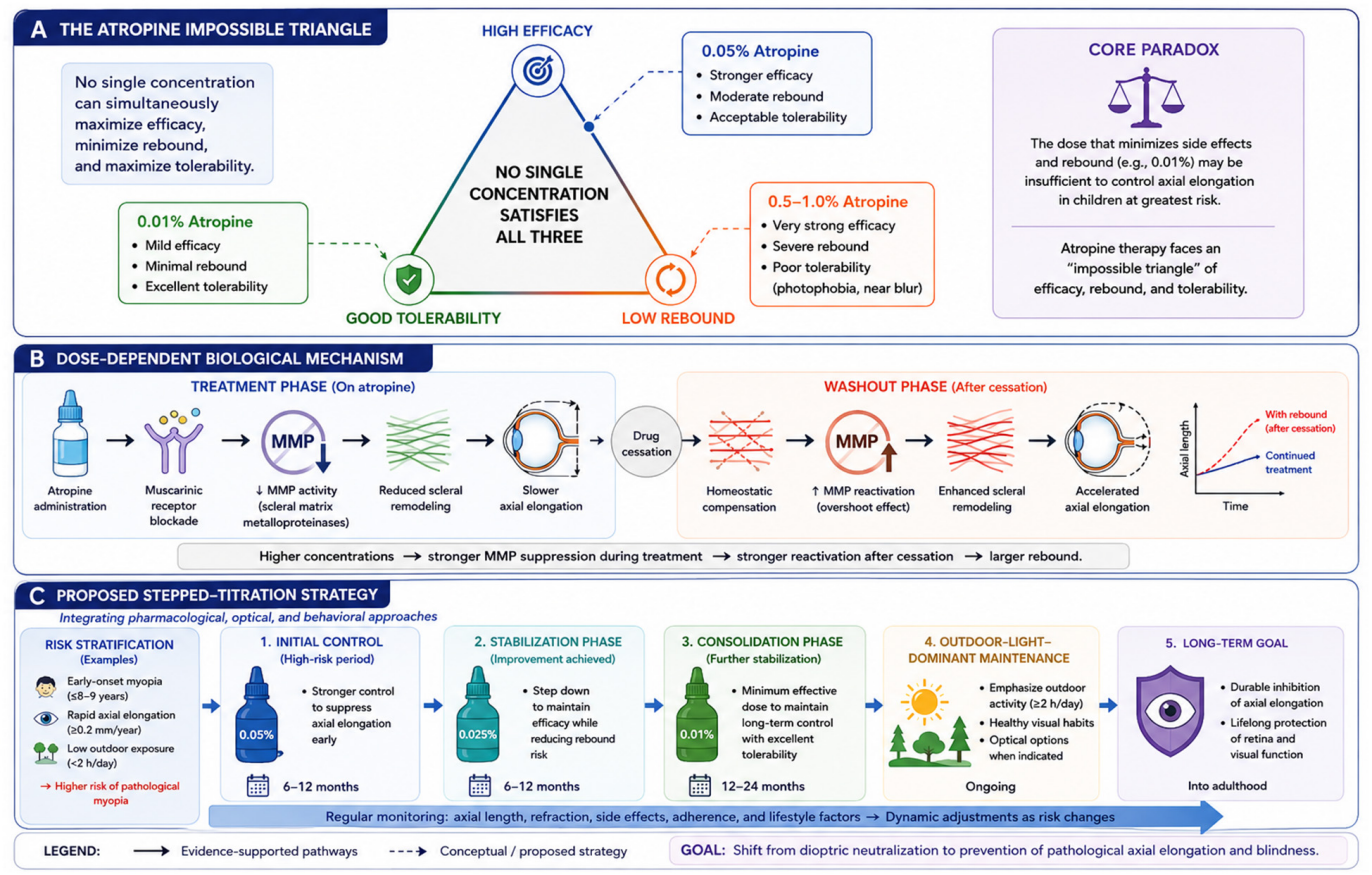
Conceptual framework of the atropine paradox in myopia control and the proposed stepped-titration strategy

Despite these advances, the narrative of low-dose superiority remains incomplete and reveals a profound clinical paradox (9). The 0.01% concentration performs well in first-year outcomes of tolerability and exhibits a reduced rebound effect upon cessation (10). Its long-term efficacy, however, deteriorates in subsequent years (11). Data from the ATOM 2 and Low-Concentration Atropine for Myopia Progression (LAMP) studies document a notable second-year decline in effectiveness, particularly for axial length control—the metric of greatest public health significance (10). Over a multi-year treatment horizon, 0.01% atropine proves markedly inferior to 0.05% atropine in restraining vitreous chamber elongation (10). Moreover, the dose-dependent rebound phenomenon, characterized by accelerated axial growth after treatment discontinuation, is not eliminated but merely attenuated at the lowest dose (11). This residual rebound may partially erode the accumulated therapeutic benefit (11). These findings expose the inherent limitations of a uniform concentration strategy applied to a heterogeneous patient population. Current practice thus oscillates between two shortcomings: higher concentrations impose an unacceptable rebound penalty and intolerable side effects, while the ostensibly benign 0.01% concentration risks therapeutic inadequacy against the primary blinding endpoint for children at the highest risk (9–11). This paradox demands a fundamental reconceptualization of atropine therapy. The path forward lies not in a single static agent but in a dynamic, risk-stratified framework where dose, treatment duration, and tapering protocols are individualized according to the patient's baseline age and axial elongation trajectory. The conceptual relationships among dose-dependent efficacy, rebound dynamics, biological mechanisms, and the proposed stepped-titration strategy are summarized in Figure 1.

## 2 Dose-dependent efficacy and rebound effect: neuroregulatory mechanisms

### 2.1 Pharmacological basis of bidirectional action

The mechanism by which atropine controls myopia extends well beyond the traditional explanation of ciliary muscle paralysis and loss of accommodation (12). Current evidence indicates that atropine acts through a bidirectional pharmacological pathway (13). As a non-selective muscarinic antagonist, it binds to muscarinic receptor subtypes located not only in the ciliary body but also across the retina, retinal pigment epithelium, choroid, and sclera (13). These posterior segment tissues are now recognized as the principal sites of action, where muscarinic signaling regulates the molecular cascade controlling eye growth (12, 13). During treatment, low-concentration atropine suppresses scleral extracellular matrix remodeling by downregulating the activity of matrix metalloproteinases (MMPs) and reducing the expression of growth factors such as transforming growth factor-beta (13, 14). This inhibitory effect is thought to contribute to the suppression of extracellular matrix remodeling associated with axial elongation. However, the precise downstream regulatory pathways involved remain incompletely understood. Yet this very mechanism creates an inherent vulnerability. Once treatment ceases, the prolonged suppression of proteolytic pathways is reversed, leading to a rebound upregulation of MMPs activity and a resurgence of scleral remodeling (14–16). The mechanistic divergence between treatment-phase inhibition and post-cessation reactivation of these enzymatic cascades forms the biological basis of the clinical rebound phenomenon and distinguishes the behavioral profiles of different atropine concentrations.

### 2.2 Asymmetry between concentration and rebound intensity

Clinical evidence from landmark trials, particularly the ATOM and LAMP studies, demonstrates a marked asymmetry between atropine concentration and rebound magnitude (15, 16). Higher concentrations, specifically 0.1% to 1.0%, produce potent suppression of axial elongation during active treatment but trigger a rapid and aggressive rebound upon discontinuation (15). This manifests as a catch-up phenomenon, wherein the elongation rate exceeds that of untreated age-matched controls, thereby canceling a substantial portion of the accumulated therapeutic gain (15). In contrast, 0.01% atropine elicits a more gradual and attenuated rebound, a feature that initially favored its widespread adoption (15, 16). However, this advantage is offset by a critical shortcoming: in younger children with aggressive progression trajectories, the 0.01% concentration does not provide sufficient suppression of axial elongation during the treatment period itself (16, 17). The LAMP study mapped this relationship through a graded dose-response curve, showing that while rebound severity scales with concentration, only moderate doses such as 0.05% deliver the sustained therapeutic effect required for high-risk populations (16, 17). This asymmetric risk profile creates a clinical dilemma: the selected concentration predetermines whether the principal failure mode will be a severe post-treatment rebound or a fundamental lack of on-treatment efficacy. The comparative characteristics of currently used atropine concentrations are summarized in Table 1.

**Table 1:** Comparative characteristics of different atropine concentrations in myopia control.

Concentration	Axial elongation control	Rebound severity	Tolerability	Suitable population	Major limitation
0.01%	Mild	Low	Excellent	Low-risk/older children	Insufficient efficacy
0.025%	Moderate	Moderate	Good	Intermediate-risk children	Limited long-term evidence
0.05%	Strong	Moderate	Acceptable	High-risk children	Potential photophobia
0.5–1.0%	Very strong	Severe	Poor	Rare severe cases	Major adverse effects

### 2.3 Washout period and its public health implications

The rebound effect following atropine discontinuation should not be interpreted as a simple biochemical withdrawal response (15, 18). It constitutes a biologically meaningful, compensatory acceleration of ocular growth with significant consequences for treatment strategy (17, 18). During the critical window of visual development, atropine pharmacologically imposes a deceleration of the eye's genetically programmed expansion trajectory (18). Once this pharmacological constraint is removed, the regulatory feedback systems governing emmetropization activate a homeostatic catch-up response, driving rapid axial elongation to compensate for the growth temporarily suspended during treatment (18). This understanding reframes the washout period not as a passive drug clearance phase but as an active, biologically driven interval of homeostatic correction. The public health significance of this reconceptualization is profound. A poorly designed cessation protocol that ignores this biological drive may provoke a period of accelerated elongation that disproportionately raises the long-term risk of pathological myopia (15, 18). This insight challenges the practice of abrupt treatment discontinuation and supports the necessity of tapered weaning regimens, age-dependent cessation timing, and seamless transition to alternative or combination therapies (15, 19, 20). The overarching therapeutic goal should therefore evolve from simply delaying myopia onset to providing durable, lifelong structural protection of the retina and optic nerve.

## 3 Reconstructing the risk–benefit assessment framework for atropine therapy

### 3.1 Redefining the “effective responder”

The conventional definition of an atropine treatment responder—a child demonstrating less than 0.5 dioptres of myopia progression per year—is both methodologically limited and clinically misleading (21). This criterion, based solely on cycloplegic refraction, overlooks the structural and functional parameters that determine long-term visual prognosis (21, 22). A clinically rigorous definition should integrate a composite set of outcome measures. Axial length elongation is the most critical parameter, as it directly quantifies the pathological process underlying irreversible vision loss (21, 22). Changes in pupil diameter may serve as a practical pharmacodynamic indicator of muscarinic blockade activity and treatment tolerability. However, current evidence does not establish pupil dilation itself as an independent predictor of long-term axial length control across different atropine concentrations (7, 21). Accommodative facility, which measures the eye's ability to shift focus rapidly, provides insight into the functional burden imposed by treatment (7). A genuinely effective responder should exhibit not merely slowed refractive change but a decelerated axial elongation trajectory aligned with age-appropriate physiological growth curves, a manageable increase in pupil size that preserves near visual function, and maintained accommodative dynamics. This multidimensional framework would enable early detection of children who satisfy the simplistic refractive criterion yet continue to experience unchecked axial progression relative to their age. Such a profile signals an elevated long-term risk of pathological myopia and demands timely therapeutic adjustment (21, 22).

### 3.2 Age and outdoor activity as core modulating variables

Age at treatment initiation and time spent outdoors represent two powerful effect modifiers that remain inadequately incorporated into current treatment algorithms (23, 24). Younger children, particularly those aged six to eight years, present a distinct biological challenge (10, 23). During this developmental phase, the eye undergoes rapid physiological growth, and the regulatory mechanisms governing emmetropization exhibit heightened plasticity (22, 25). Low-concentration atropine (0.01%) frequently proves insufficient to counteract the strong endogenous drive for axial elongation in this age group, placing these children at substantial risk of undertreatment (10, 21). The therapeutic window is narrowest when the potential for cumulative structural damage is greatest. Concurrently, environmental light exposure offers a synergistic mechanism that may help resolve this impasse (24, 26). Bright outdoor light stimulates retinal dopamine release, a well-established inhibitor of axial elongation operating through a pathway independent of muscarinic antagonism (25, 26). Combining atropine therapy with high levels of outdoor activity therefore constitutes a dual-mechanism strategy: pharmacological suppression of scleral remodeling alongside light-mediated, dopamine-driven growth inhibition (24–26). This synergy holds the potential to overcome the limitations of low-dose monotherapy by reinforcing therapeutic efficacy through a complementary biological pathway.

### 3.3 Public health economic considerations of combination therapy

In resource-limited public health systems, the strategic allocation of screening and treatment resources demands cost-effectiveness analysis. A monotherapy model using a single, fixed-concentration atropine regimen incurs economic burdens that extend well beyond drug acquisition (15, 21). These include the costs of frequent follow-up visits, management of rebound-related treatment failures, and long-term care for preventable pathological complications (15, 27). Against this backdrop, a progressive combination strategy merits serious consideration as a potentially superior approach. Initiating treatment with a moderate concentration such as 0.05% atropine, combined with optical interventions like peripheral defocus lenses, engages two complementary mechanisms: pharmacological and optical pathways simultaneously targeting the biochemical and optical triggers of axial elongation (28, 29). This combined approach may facilitate a more gradual and biologically stable weaning process. However, definitive evidence regarding its ability to shorten overall treatment duration remains limited and requires validation through long-term prospective studies (15, 28). From a health economics standpoint, the higher initial investment in combined therapy could be offset by decreased aggregate follow-up requirements, fewer repeat treatment cycles, and a lower incidence of end-stage myopic complications necessitating costly tertiary care (15, 27, 30). Such a framework realigns short-term expenditure with long-term societal benefit, transforming myopia management from a reactive, serial process into a coordinated, risk-stratified intervention designed to maximize structural protection of the posterior segment.

## 4 Future directions and recommendations

### 4.1 From a fixed concentration to a stepped-titration approach

The evidence opposing a single-concentration strategy for all patients compels a shift toward dynamic, risk-stratified treatment algorithms (15, 28). A clinically sound framework would calibrate the initial atropine dose against a composite risk profile that includes baseline axial length, age, and daily outdoor time. Key variables incorporated into this personalized risk-stratified framework and their corresponding therapeutic implications are summarized in Table 2. Children with significantly elongated axial length for their age, early onset of myopia, and minimal environmental protection constitute the highest-risk group (24, 28). For these patients, initiating treatment with a moderate concentration such as 0.05% is appropriate to achieve rapid and decisive control of the elongation trajectory (16, 28). Once axial growth stabilizes within age-appropriate physiological limits, a structured de-escalation can begin (15, 16). The concentration would be tapered progressively toward 0.025% and eventually 0.01%, with the explicit aim of shifting the primary mode of control from pharmacological suppression to environmental reinforcement (15, 24). During this later phase, outdoor exposure would be deliberately intensified to harness the inhibitory effect of light-stimulated retinal dopamine release (24, 25). This stepped-titration model reframes atropine not as chronic maintenance therapy but as a time-limited intervention bridging a vulnerable developmental window. Sustained long-term protection may increasingly depend on behavioral and environmental mechanisms once pharmacological intensity is reduced.

**Table 2:** Key components of a personalized risk-stratified atropine treatment framework.

Clinical variable	Risk implication	Therapeutic adjustment
Younger age	Faster axial growth	Higher initial concentration
Rapid axial elongation	High pathological risk	Aggressive control
Low outdoor exposure	Reduced dopamine- mediated protection	Behavioral reinforcement
Strong rebound history	Washout vulnerability	Slower tapering
Poor tolerability	Adherence risk	Dose reduction

### 4.2 Digital monitoring and cessation decision-making

The precision of atropine therapy is fundamentally limited by the inability to quantify real-world treatment adherence and environmental exposures that critically influence outcomes (31, 32). Wearable light-sensing technologies offer a promising solution to this challenge (31, 32). These devices can continuously record spectral composition, illuminance levels, and wear-time, generating high-resolution data on actual outdoor exposure in place of imprecise parental reports. When combined with emerging or conceptual systems designed to estimate treatment adherence and drug administration behavior, such data may support the construction of an integrated therapeutic record (21, 32). This platform could capture the interplay between pharmacological adherence, environmental light dose, and serial axial length measurements, allowing predictive algorithms to identify the optimal moment for dose reduction. The central clinical imperative is to avoid abrupt, calendar-driven cessation that disregards individual biological readiness and triggers a cliff-edge rebound with lasting structural consequences (15, 21). A data-driven cessation model grounded in objective signals of ocular growth stabilization and sufficient environmental protection would represent a significant advance toward personalized myopia care, transforming a reactive process into an anticipatory and adaptive intervention.

### 4.3 The urgent need for multicentre real-world data

A significant evidence gap remains between the controlled conditions of randomized trials and the heterogeneous reality of clinical practice (21, 22). Current pivotal studies have largely reported efficacy during active treatment, with rebound outcomes often confined to secondary analyses or limited by short observation periods (15, 16).

This reporting asymmetry may inadvertently overestimate the net therapeutic benefit, partly because prolonged pediatric follow-up after treatment cessation is often constrained by practical and logistical challenges (15, 21). A decisive corrective is required: the magnitude and time course of rebound should be elevated to a mandatory, co-primary endpoint in all future atropine trials for myopia control. Study designs should mandate a washout observation period of no less than two to three years following treatment discontinuation, with standardized serial measurements of axial length and refractive error (15, 16, 21). Only through such extended follow-up can the true net efficacy—the residual treatment benefit after complete rebound resolution—be accurately determined (15, 21). Furthermore, multicentre real-world data registries that capture diverse populations, practice patterns, and environmental contexts are essential to validate the generalizability of stepped-titration protocols and to characterize the long-term safety profile of prolonged muscarinic antagonism in the developing eye (21, 22). Such evidence would constitute the foundation for evidence-based clinical guidelines that transcend the limitations of individual trials and align therapeutic recommendations with the overarching public health objective of blindness prevention.

## 5 Summary

The atropine paradox is, at its core, a conflict between two clinical philosophies: treating the drug as a simple topical agent for refractive control, or deploying it as a developmental modulator that fundamentally alters the trajectory of ocular growth. Resolving this tension requires an unequivocal reclassification of atropine into the latter category. Effective myopia control cannot be achieved through pharmacological optimization in isolation, detached from the environmental determinants that shape refractive development. Any clinically coherent and publicly responsible strategy should therefore elevate outdoor activity and the reduction of intensive near work from ancillary recommendations to co-equal, evidence-based interventions embedded within adolescent health policy. This approach acknowledges that biochemical and environmental pathways converge upon the scleral remodeling cascade and should be addressed in concert.

A decisive consensus should now be forged between ophthalmic clinicians and public health decision-makers. The optimal atropine concentration is not the one that yields the most potent short-term suppression of myopia progression. Rather, it is the concentration that safely guides the child through the period of rapid axial elongation while minimizing disruption to the natural rhythms of visual development. The ultimate measure of therapeutic success is not a refractive value captured at an isolated time point, but the lifelong structural integrity of the retina and optic nerve. This reconceptualization shifts the goal of myopia management from the temporary stabilization of dioptric measurements to the durable prevention of blindness.

## Data Availability

The original contributions presented in the study are included in the article/supplementary material, further inquiries can be directed to the corresponding author.
